# Nano silica diaphragm in-fiber cavity for gas pressure measurement

**DOI:** 10.1038/s41598-017-00931-0

**Published:** 2017-04-11

**Authors:** Shen Liu, Yiping Wang, Changrui Liao, Ying Wang, Jun He, Cailing Fu, Kaiming Yang, Zhiyong Bai, Feng Zhang

**Affiliations:** Key Laboratory of Optoelectronic Devices and Systems of Ministry of Education and Guangdong Province, College of Optoelectronic Engineering, Shenzhen University, Shenzhen, 518060 China

## Abstract

We demonstrate an ultrahigh-sensitivity gas pressure sensor based on the Fabry-Perot interferometer employing a fiber-tip diaphragm-sealed cavity. The cavity is comprised of a silica capillary and ultrathin silica diaphragm with a thickness of 170 nm, with represents the thinnest silica diaphragm fabricated thus far by an electrical arc discharge technique. The resulting Fabry-Perot interferometer-based gas pressure sensor demonstrates a gas pressure sensitivity of about 12.22 nm/kPa, which is more than two orders of magnitude greater than that of a similarly configured fiber-tip air bubble sensor. Moreover, our gas pressure sensor has a low temperature cross-sensitivity of about 106 Pa/°C, and the sensor functions well up to a temperature of about 1000 °C. As such, the sensor can potentially be employed in high-temperature environments.

## Introduction

Various types of fiber optic sensors, such as long period fiber gratings^1–3^, fiber Bragg gratings^4–6^, Mach-Zehnder interferometers (MZIs)^7–9^, and Fabry-Perot interferometers (FPIs)^10–27^, have been reported to fulfill specific gas pressure application. Among the above gas pressure sensors, FPI-based sensors have played a dominant role due to their capacity for miniature size and ultra-high sensitivity. Gas pressure sensors based-on fiber-optic FPIs have been widely used in various fields such as the automotive industry, environmental monitoring, biomedicine, voice communication, and nondestructive health monitoring. A silica-based FPI gas pressure sensor fabricated at the tip of an optical fiber has the advantages of excellent thermal stability, high sensitivity, compact size, and immunity to electromagnetic interference, enabling a robust functionality in harsh physical and chemical environments^28, 29^.

Currently, FPI-based gas-pressure sensor can be divided into two types: one type that employs an opened cavity and the other that employs a hermetic cavity sealed with a thin flexible diaphragm. For the first types of sensor, the refractive index (RI) of the gas in the open cavity varies with changing gas density according to the gas pressure. For instance, an open-cavity optical fiber FPI based on dual capillaries exhibited a wavelength shift sensitivity of about 4.1 nm/MPa^11^. Here, the wavelength shift sensitivity is denoted as the ratio of an observed wavelength shift to a given change in gas pressure. In addition, Quan *et al*. proposed an optimized structure based on a photonic crystal fiber and the Vernier effect, which further enhanced the wavelength shift sensitivity to about 82 nm/MPa^12^. However, because the RI generally varies according to the gas composition, the measurement accuracy of an open cavity FPI-based gas pressure sensor is affected signally by the gas composition in the environment. For the second type of FPI-based gas pressure sensor, the length of the hermetic cavity varies according to the external gas pressure via the induced deflections of the sensing diaphragm. Here, the gas pressure sensitivity is defined as the ratio of the cavity length variation to changes in the external gas pressure. For example, FPI-based gas pressure sensors employing a polymer-metal composite diaphragm^13^, a grapheme diaphragm^14^, and a sliver diaphragm^15^ have exhibited ultrahigh gas pressure sensitivity of about 1.54, 39.4 and 70.5 nm/kPa, respectively. Unfortunately, the structural robustness or high-temperature performance of these FPI-based sensors is poor, which limits their application in harsh environments. Wang *et al*. demonstrated an FPI-based gas pressure sensor employing a hermetic cavity and a moveable liquid level with a gas pressure sensitivity >1000 nm/kPa. However, the properties of the liquid level are greatly influenced by the temperature, and it exhibits a larger thermal expansion coefficient than either silicon or silica, so that the temperature induced cavity length change at atmospheric pressure is about 333 nm/°C^16^.

All-silica FPI-based gas pressure sensors employing a hermetic cavity with a sensing diaphragm have been widely developed because of their wide range of working temperatures and high-temperature stability. A number of fabrication methods for creating all-silica sensors have been reported. For example, A. Wang’s group demonstrated a series of all-silica FPI-based pressure sensors, including a sensor employing a thin fused silica diaphragm at the end of a ferrule^17^, a miniature sensor fabricated on the tip of an optical fiber^18^, and a high-temperature fiber-tip pressure sensor^20^. All of the sensors were fabricated by use of the splicing-and-cutting method, and their sensing diaphragm thicknesses were further reduced by means of HF etching. In addition, the thickness of the sensing diaphragm has also been controlled by pre-polishing and in-line monitoring during HF etching^21, 22^. Wang *et al*. fabricated an all-silica ultrathin diaphragm of uniform thickness at the end of an FPI by means of a silicon oxide layer and the HF etching method, which provided for enhanced gas pressure sensitivity^23^. While the fabrication of sensors discussed above in this paragraph employed hazardous chemical etching methods for reducing the thickness of the sensing diaphragm, alternative methods of diaphragm thinning have also been reported, such as a femtosecond (fs) laser micromachining method^24^ and the employment of a fusion splicer and a pressurizing gas chamber^25^, although the equipment costs are generally greater than that associated with chemical etching. Recently, Liu *et al*. demonstrated a promising electrical arc-discharge technique to create an air-cavity FPI-based gas pressure sensor in optical fiber^26^, and this technique was further optimized to fabricate an FPI-based sensor employing a fiber-tip air bubble (FAB)^27^. This fabrication method is very simple, and requires only a commercial fusion splicer without additional pressurization equipment. Unfortunately, the gas pressure sensitivity is poor due to the special sphere-shape of the sensing diaphragm at the end of the FAB.

In this paper, we present a novel method employed for fabricating an all-silica gas pressure sensor based on an FPI with a fiber-tip diaphragm-sealed cavity (FDC). This sensor employing an ultrathin all-silica sensing diaphragm with a thickness of about 170 nm has been achieved by means of an optimized electrical arc-discharge technique. To authors’ best knowledge, this sensor currently employs the thinnest all-silica diaphragm to have been incorporated into an FPI-based gas pressure sensor fabricated by an electrical arc discharge technique. Furthermore, the direct thermal splicing between the ultrathin all-silica diaphragm and the end face of an optical fiber using only a commercial fusion splicer has not been previously reported. The resulting gas pressure sensor demonstrates good thermal stability and ultrahigh sensitivity. The gas pressure sensitivity of the sensor with a cavity length of about 55 µm and a diaphragm thickness of less than 250 nm was measured to be 12.22 nm/kPa, which is more than two orders of magnitude greater than that of FPI-based sensors employing an FAB. Moreover, the sensor was measured at a high-temperature of 1000 °C, and the cavity length temperature sensitivity was about 1300 pm/°C, whereas the temperature-induced gas pressure measurement error was less than 106 Pa/°C.

## Experiments

**Figure 1:**
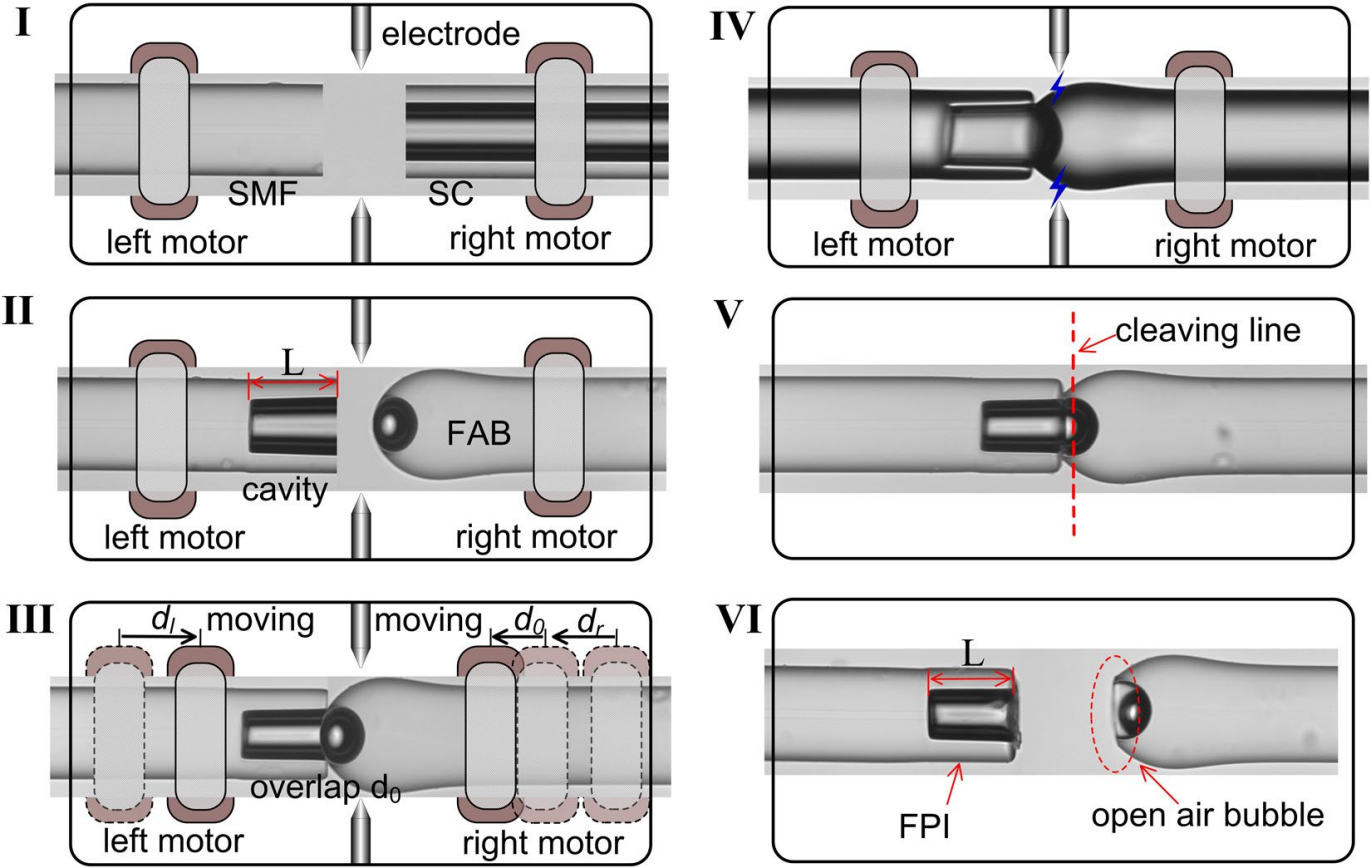
Schematic diagram of the fabrication process for an FPI-based gas pressure sensor employing an FDC using electrical arc discharge in conjunction with single mode fiber (SMF), silica capillary (SC), and fiber-tip air bubble (FAB) components.

Figure 1 illustrates the fabrication process of an in-fiber FPI employing an FDC, which involves four steps. In step 1, a standard 125 µm single mode fiber (SMF) is spliced to a section of a silica capillary (SC) having an inner diameter of 75 µm and an outer diameter of 125 µm by means of a commercial fusion splicer, as shown in the Fig. 1(I). The spliced configuration was then cleaved, and the well-cleaved end of the SC of length L was placed in the left fiber holder, and a previously prepared FAB with a nano silica diaphragm fabricated by a repeating arc discharge method reported in ref. 27 was placed in the right fiber holder, as shown in Fig. 1(II). In step 2, as shown in Fig. 1(III), the left and right fiber ends were moved toward each other until achieving contact between the cleaved capillary end and the FAB by carefully controlling the movement of the left and right motors of the fusion splicer, denoted as d_l_ and d_r_, respectively. After achieving contact, the right holder was subsequently moved an additional distance d_0_, resulting in an overlap of d_0_ at the point of contact, which applied a smaller axial stress to the region of contact. It should be noted that the value of d_0_ here is no more than a few micrometers to avoid damaging the tip of the FAB, compared to the reported in ref. 10. In step 3, as shown in Fig. 1(IV), a precise electrical arc discharge with a fusion current of about 18 mA and a fusion time of about 500 ms was applied at the point of contact. As a result, the silica wall of the FAB and the cleaved capillary end were heated to a melted state, and were effectively spliced together under the pre-applied axial stress. Because the external surfaces of the silica materials of the capillary and FAB respectively soften and solidify more rapidly than the internal surfaces during and after the short period of arc discharge, the tip of the FAB at the point of contact is easily spliced to the end of the capillary, forming the diaphragm, and the quality of the internal surface of the sensing diaphragm is not affected. In step 4, the precision cleaving configuration consists of an optical fiber cleaver, a CCD camera (http://www.twsunway.com), and a precision displacement platform with a step of 0.1 µm. The CCD camera was employed to monitor the desired cleaving line, the cleaving edge of an optical fiber cleaver, and the spliced region between the cleaved capillary end and the FAB. As shown in Fig. 1(V), the spliced region was accurately cleaved along the desired cleaving line indicated by a red dotted line in the figure. In our experiments, the difference between the achieved and desired cleaving lines was smaller than ±1 µm, which indicates our cleaving process has a very good repeatability. As a result, an FPI-based gas pressure sensor with a cavity length of L was achieved, as shown in Fig. 1(VI).

**Figure 2:**
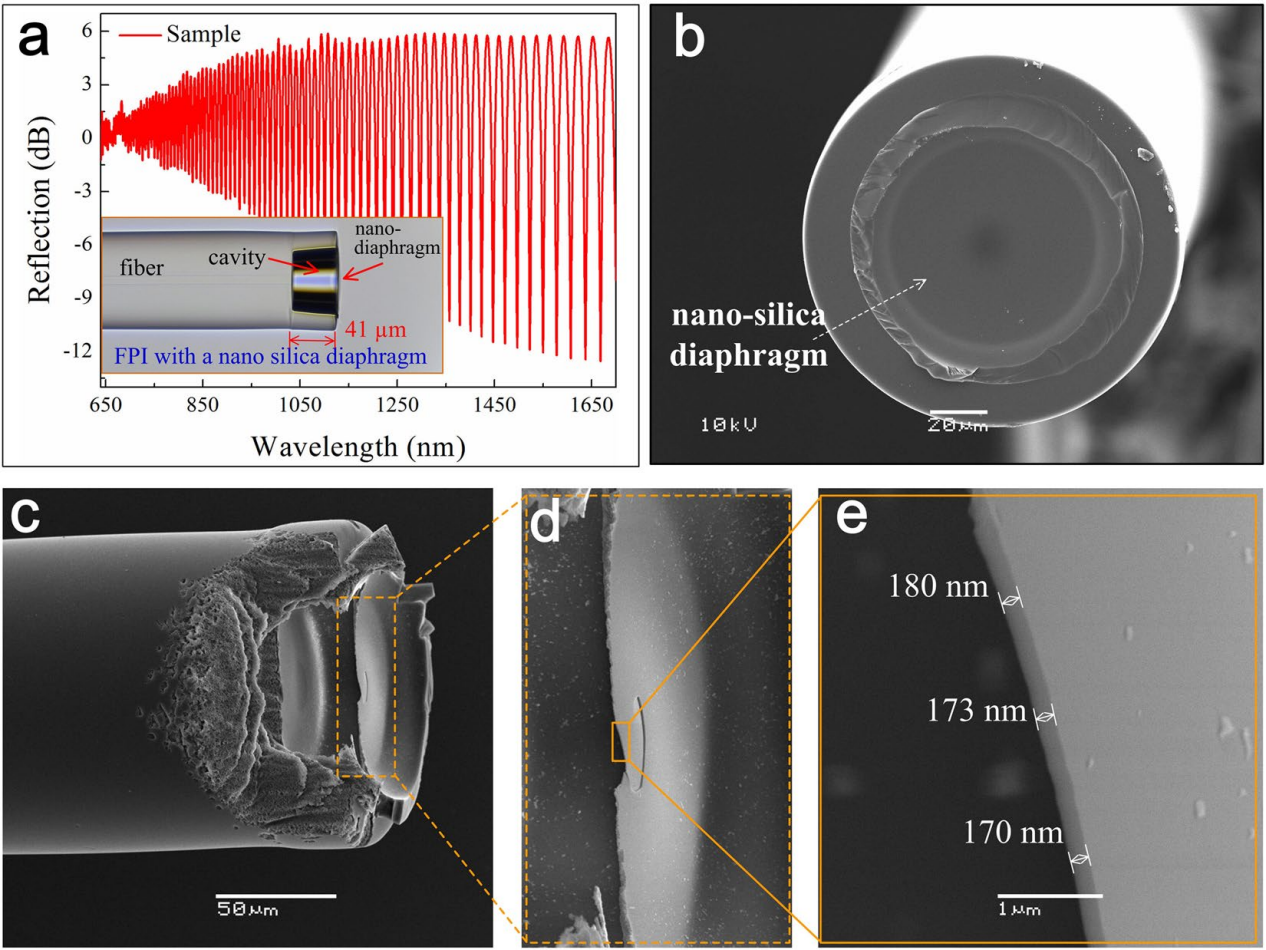
The reflection spectrum (**a**) and SEM images (**b–e**) of an FPI-based gas pressure sensor specimen employing an FDC. The inset of (**a**) presents an optical microscopy image. (**b**) SEM image of the nanometer-scale all-silica diaphragm fused at the end of the silica capillary. (**c**) SEM image of a fractured end of the FDC, while (**d,e**) present enlarged SEM images of the silica diaphragm, showing a sensing diaphragm thickness of about 170 nm.

Figure 2(a) presents the reflection spectrum of an FPI-based sensor specimen employing an FDC, and a corresponding optical microscopy image is provided as an inset. The reflection spectrum was obtained using a broad-band light source (NKT Photonics SuperK Compact, wavelength range from 500 to 2400 nm), an optical spectrum analyzer (YOKOGAWA AQ6370C) with a resolution of 0.02 nm, and a 3-dB fiber coupler. The length of the FDC was estimated from the optical microscopy image to be about 41 µm, and the thickness of the sensing diaphragm was no more than 180 nm, as calculated from the envelope spacing of the reflection spectrum (more than 2120 nm). Figure 2(b) presents a scanning electron microscopy (SEM) image of the sensing diaphragm at the end of the SC. For demonstrating the thickness of the sensing diaphragm more clearly, an SEM image of a sensing diaphragm fractured by fs laser micromachining is shown in Fig. 2(c). Figure 2(e) presents an enlarged cross-sectional view of the sensing diaphragm, where the thickness of the thinnest region of the diaphragm is measured to be about 170 nm, to authors’ best knowledge, which is the thinnest all-silica diaphragm fabricated by an electrical arc discharge technique.

### Gas pressure response

**Figure 3:**
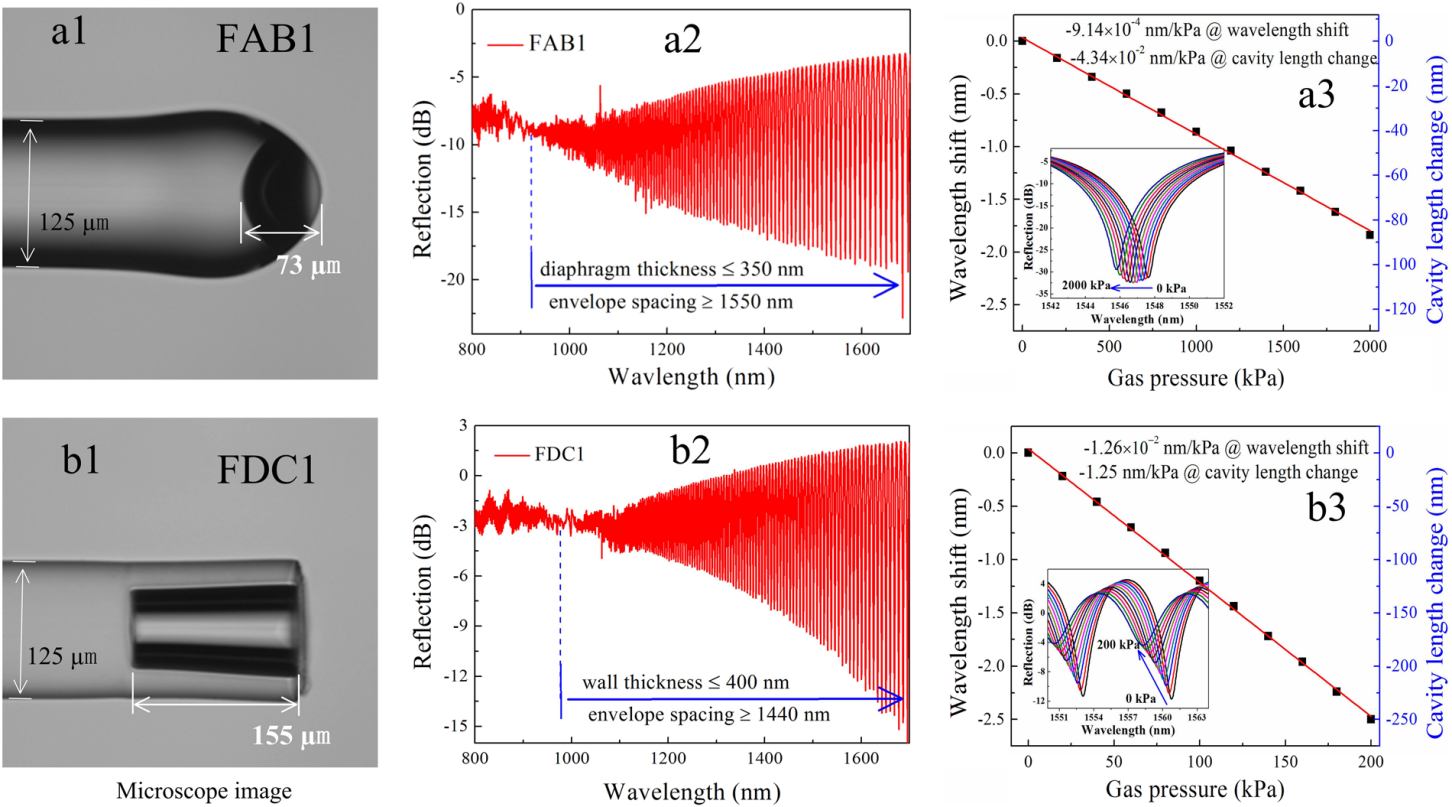
Pressure responses of two types of FPI-based gas pressure sensor specimens employing an FAB or an FDC. (**a1**) Optical microscopy image of specimen FAB1 employing an FAB with a diaphragm thickness of about 350 nm, as calculated from the envelope spacing of its reflection spectrum given in (**a2**). (**a3**) Wavelength shift of the interference fringe around 1550 nm with the gas pressure varying from 0 to 2.0 MPa, and the slope of the linear fitting is about 9.14 × 10^−4^ nm/kPa, reflecting a corresponding gas pressure sensitivity of about 4.34 × 10^−2^ nm/kPa. The inset presents the reflection spectral evolution. (**b1**) Optical microscopy image of specimen FDC1 employing an FDC with a diaphragm thickness of about 400 nm, as calculated from the envelope spacing of its reflection spectrum given in (**b2**). (**b3**) Wavelength shift of the interference fringe around 1550 nm with the gas pressure varying from 0 to 0.2 MPa, and the gas pressure sensitivity is about 1.25 nm/kPa. The inset presents the reflection spectral evolution.

Figure 3(a1) presents an optical microscopy image of an FAB with an air cavity length estimated to be about 73 µm, denoted as specimen FAB1, that was fabricated by a previously reported method^27^. The sensing diaphragm thickness of FAB1 was estimated from the envelope spacing (more than 1660 nm) of the reflection spectrum shown in Fig. 3(a2) to be no more than 350 nm. The measured fringe contrast of FAB1 was about 22 dB, and the fringe spacing was about 15.5 nm at around 1550 nm under standard atmospheric pressure and room temperature conditions. FAB1 was sealed in a gas chamber, and the pressure response was measured under conditions where the gas pressure was increased from 0 to 2.0 MPa in increments of 0.2 MPa, remaining at each step for 10 min. The reflection spectrum evolution at different pressure conditions is shown in the inset of Fig. 3(a3), and a linear fitting to the experimental data provides a slope of 9.2 × 10^−4^ nm/kPa for FAB1. From this data, the gas pressure sensitivity, given as the ratio of the cavity length variation to changes in the gas pressure, is determined to be 4.34 × 10^−2^ nm/kPa. It can be seen that, when the pressure increases, the interference dip shifts toward shorter wavelengths. For static gas pressure sensing, the relationship between the cavity length change ∆L and the wavelength shift ∆λ can be described by ${\rm{\Delta }}L=L{\rm{\Delta }}\lambda /\lambda$, which represents the deflection variation of the sensing diaphragm as a result of gas pressure changes^14^.

Using the fabrication process illustrated in Fig. 1, and employing FAB1 as the FAB in the process, the FDC-based gas pressure sensor, denoted as FDC1, shown in Fig. 3(b1) was fabricated with a cavity length of about 155 µm and a diaphragm thickness at the end of the SC of no more than 420 nm, as calculated from the envelope spacing (more than 1400 nm) of the reflection spectrum shown in Fig. 3(b2). Under equivalent test conditions as those employed for FAB1, the reflection spectrum evolution of FDC1 at different external gas pressures is shown in the inset of Fig. 3(b3), and the slope of a linear fitting was determined to be about 1.26 × 10^−2^ nm/kPa about 1550 nm with a corresponding gas pressure sensitivity of about 1.25 nm/kPa. As a result, the gas pressure sensitivity of FDC1 is more than one order of magnitude greater than that of FAB1.

### Enhancing gas sensitivity by optimizing sensor diaphragm

**Figure 4:**
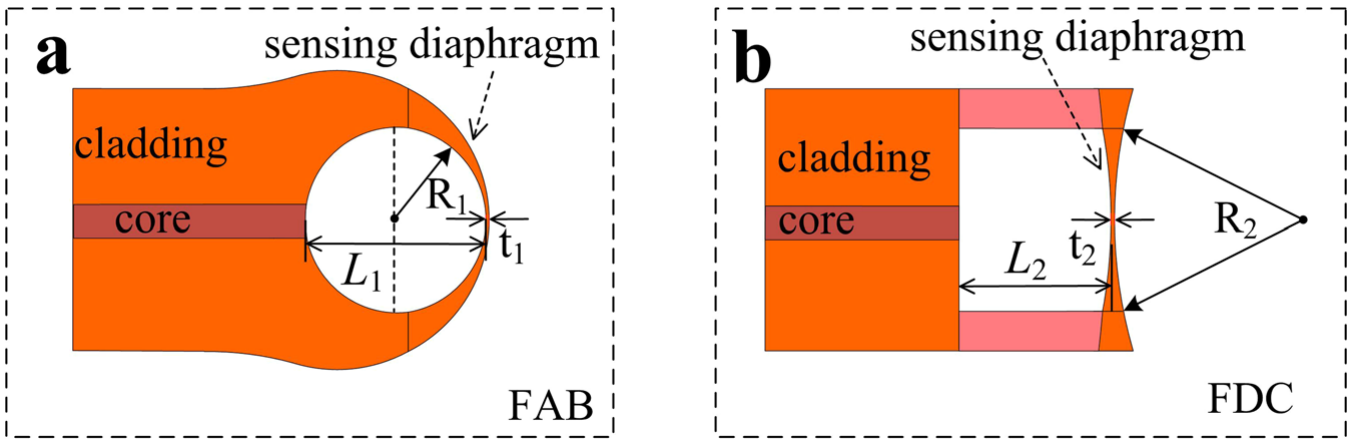
Schematic diagrams illustrating the geometric parameters affecting the gas pressure sensitivity. (**a**) An FPI-based gas pressure sensor employing an FAB. (**b**) An FPI-based gas pressure sensor employing an FDC.

Approximately, the cavity length change Δ*L* may be related to the gas pressure change Δ*p* by ref. 25,

1 $${\rm{\Delta }}L=\frac{(1-\nu ){R}^{2}}{2E\,t}\,{\rm{\Delta }}P={S}_{p}\,{\rm{\Delta }}P,$$

where *v* is Poisson’s ratio, R is the radius of the spherical cavity, *E* is the Young’s modulus of silica, t is the diaphragm thickness, and S_p_ is the sensor’s gas pressure sensitivity. The geometrical parameters for gas pressure sensors based on FAB and FDC configurations are illustrated in Fig. 4.

According to Eq. (1), S_p_ can be improved by either increasing R or by reducing t. The effectiveness of enhancing S_p_ by reducing t has been reported previously^27^ using a repeatedly implemented electrical arc discharge at the tip of the FAB. However, the present study adopted a more effective approach for improving S_p_ by reducing the curvature of the sensing-diaphragm (i.e., 1/R). A comparison of Fig. 3(a1,b1) indicate that the curvature of the sensing diaphragm at the end of specimen FDC1 is far less than that of the tip of the FAB1 specimen, and S_p_ was consequently increased by an order of magnitude from 4.34 × 10^−2^ to 1.25 nm/kPa.

Note that Eq. (1) treats the sensing diaphragm as a thin spherical shell of uniform thickness, although the silica wall is actually not uniform, where the central region is much thinner than the surrounding area, as shown in Fig. 4(b), and this gradually-changing thickness negatively affects the gas pressure response sensitivity. Therefore, optimizing the uniformity of the diaphragm cross section can further improve S_p_. To this end, it will be noted that a much larger FAB relative to the diameter of the cavity would exhibit a smaller deviation in thickness in the region surrounding the tip, which can be exploited to fabricate a sensing diaphragm with a more uniform thickness.

**Figure 5:**
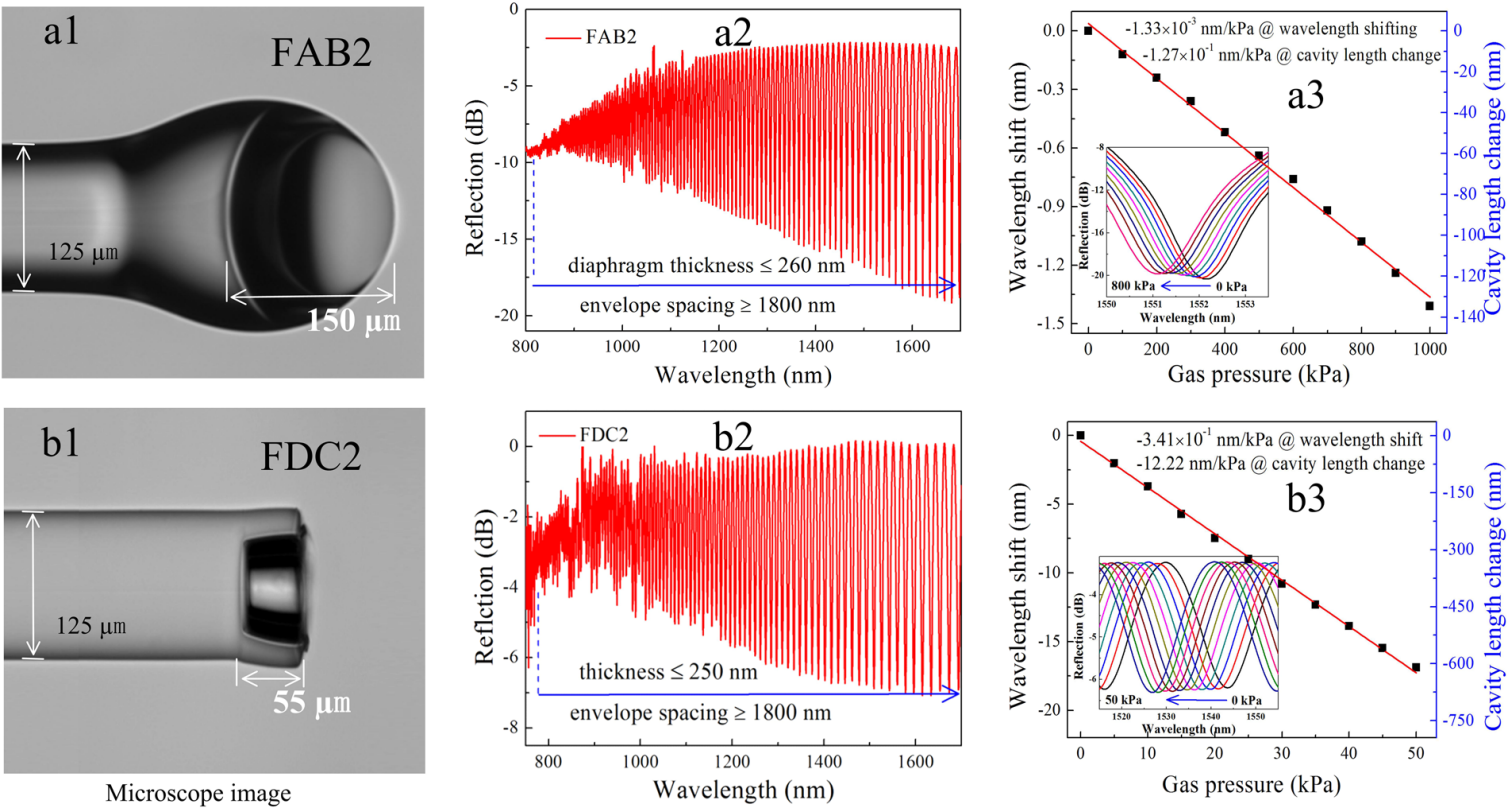
Sensor specimens based on an optimized sensing diaphragm. (**a1**) Optical microscopy image of specimen FAB2 with a cavity length of about 155 µm and a diaphragm thickness of about 290 nm. (**a2**) The reflection spectrum of FAB2. (**a3**) Wavelength shift of the interference fringe around 1530 nm with the gas pressure varying from 0 to 1.0 MPa, and the slope of the linear fitting is about 1.33 × 10^−3^ nm/kPa, reflecting a corresponding gas pressure sensitivity of about 1.27 × 10^−1^ nm/kPa. The inset presents the reflection spectral evolution. (**b1**) Optical microscopy image of specimen FDC2 employing an FDC with a cavity length of about 55 µm and a diaphragm thickness of about 250 nm. (**b2**) The reflection spectrum of FDC2. (**b3**) Wavelength shift of the interference fringe around 1530 nm with the pressure varying from 0 to 50 kPa, and the corresponding gas pressure sensitivity based on the slope of the linear fitting is about 12.22 nm/kPa. The inset presents the reflection spectral evolution.

An FDC-based gas pressure sensor, denoted as FDC2, with a cavity length of about 155 µm, as measured from the optical microscopy image shown in Fig. 5(a1), was fabricated employing the larger sized FAB, denoted as FAB2, shown in Fig. 5(b1), for enhancing S_p_. The volume of FAB2 is greater than eight times that of the previously employed FAB1 shown in Fig. 3(a1). The sensing diaphragm thickness of FAB2 was less than 260 nm, as calculated by the envelope spacing of the reflection spectrum shown in Fig. 5(a2). An enhanced electrical arc discharge, relative to the fabrication parameters employed for FAB1, with a fusion current of ~25 mA and a fusion time of ~2000 ms was employed in the FDC2 fabrication process to fuse the FAB to the cavity end. As a result, the curvature (1/R) of sensing diaphragm FAB2 is smaller than that of FAB1, and has a more uniform thickness. The gas pressure response of FAB2 was measured under an equivalent test environment as that of FAB1. As shown in Fig. 5(a3), a gas pressure sensitivity of about 1.27 × 10^−1^ nm/kPa was achieved around 1550 nm. Although FAB2 has a larger sized air bubble, the gas pressure sensitivity is not significantly greater than that of FAB1, where S_p_ only increased from 4.34 × 10^−2^ to 1.27 × 10^−1^ nm/kPa. In contrast, FDC2 exhibited an outstanding gas pressure performance, owing to a diaphragm thickness that was less than 250 nm, as calculated by the envelope spacing of the reflection spectrum shown in Fig. 5(b2), and an ultrahigh gas pressure sensitivity of about 12.22 nm/kPa around 1530 nm was achieved, as shown in Fig. 5(b3), which is nearly 100 times greater than that of FAB2. These results indicate that the optimized sensing diaphragm structure can significantly enhance S_p_.

### Temperature response

In order to estimate the gas pressure in the FDC cavity, two temperature states have been considered. The first state is a moment that the diaphragm-sealed is spliced at the end of the FDC cavity at high temperature. In the FDC cavity, the volume of FDC cavity is V_1_, an estimated air pressure is P_1_ of ~10^5^ pa, and the temperature is T_1_ of ~1800 K, less than the temperature at the electrode center, where the splicing region of FDC cavity is slightly deviated from the center of electrical arc discharge, as shown in Fig. 1(III). In the second state, we obtained a sample FDC with a hermetic cavity at room temperature when the arc discharge was finished. As shown in Fig. 1(IV), the parameters of intra FDC cavity are assumed, the gas pressure is P_2_, the volume of cavity is unchanged V_2_ = V_1_, and a temperature is T_2_ of ~300 K. Using the idea gas law, P_2_ = (T_2_/T_1_)*P_1_, i.e. P_2_ = 0.17P_1_, therefore, a temperature increase of 1 degree at room temperature (300 K) will cause a gas pressure increase of ~0.057 kPa of the trapped air in the FDC cavity. In addition, the measured gas pressure sensitivity of samples, i.e. FDC1 and FDC2, are 1.25 and 12.22 nm/kPa, respectively. Based on the above theoretical analysis using the idea gas law, the estimated cavity length change of samples, i.e. FDC1 and FDC2, are 70.76 and 696.54 pm/°C, due to the pressure change from temperature increase.

**Figure 6:**
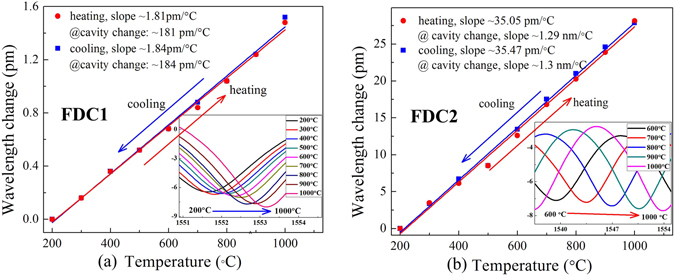
High temperature response of samples from room temperature to 1000 °C. As shown in (**a**,**b**), the corresponding to cavity change sensitivity, i.e. FDC1 and FDC2, are about 184 and 1300 pm/°C. The inset presents the reflection spectral evolution versus ambient temperature around 1550 nm.

Furthermore, the temperature responses of sensors, i.e. FDC1 and FDC2, have been also investigated in conjunction with heating in an electrical tubular-oven (Cabolite EST12/300B-230SN), which can reach the temperature as high as 1200 °C. At first, the samples were loosely placed in the electrical tubular-oven with no external stress applied. Next, the temperature was raised from room temperature to 1100 °C and kept at 1100 °C for 2 hours to make a long-term annealing of the samples. After that, the temperature response of those samples were measured, where the reflection spectrum evolution were monitored by an OSA and a light source during heating and cooling those samples. As shown in Fig. 6(a,b), the FDC1 and FDC2 were cooled form 1000 °C to 200 °C with a step of 100 °C, and then heated up to 1000 °C with the same step. The linear fitting to the wavelength change of the samples, i.e. FDC1and FDC2, provide a slope of 1.84 and 35.47 pm/°C, and corresponding to cavity change sensitivity of 184 and 1300 pm/°C, respectively. As a result, the measured cavity change sensitivity of the samples (FDC1, FDC2), is 2.59 and 1.87 times higher than that of theoretical values, i.e. 70.76 and 696.54 pm/°C, respectively. The data errors can be explained by two factors: (1) Material temperature effect (expansion and contraction), i.e. the cavity length of FDC based on silica may elongate with increasing of ambient temperature. (2) The sensing diaphragm as a thin spherical shell with a gradually-changing thickness may produce some nonlinear deformation along with the temperature change. In addition, the cavity lengths, gas pressure sensitivities, cavity length sensitivities to temperature (i.e., the ratio of the cavity length variation to temperature change), and temperature cross-sensitivities (i.e., temperature-induced gas pressure measurement error) for sensors FAB1, FDC1, FAB2, and FDC2 are summarized in Table 1.

**Table 1:** Parameter comparison of several FPIs.

Samples	Cavity length (µm)	Gas pressure sensitivity (nm/kPa)	Temperature sensitivity (pm/°C)	cross-sensitivity (Pa/°C)
FAB1	73	4.34 × 10^−2^	38.60	889.40
FDC1	155	1.25	184	147.20
FAB2	150	1.27 × 10^−1^	59	464.57
FDC2	55	12.22	1300	106.38

### Numerical simulations

**Figure 7:**
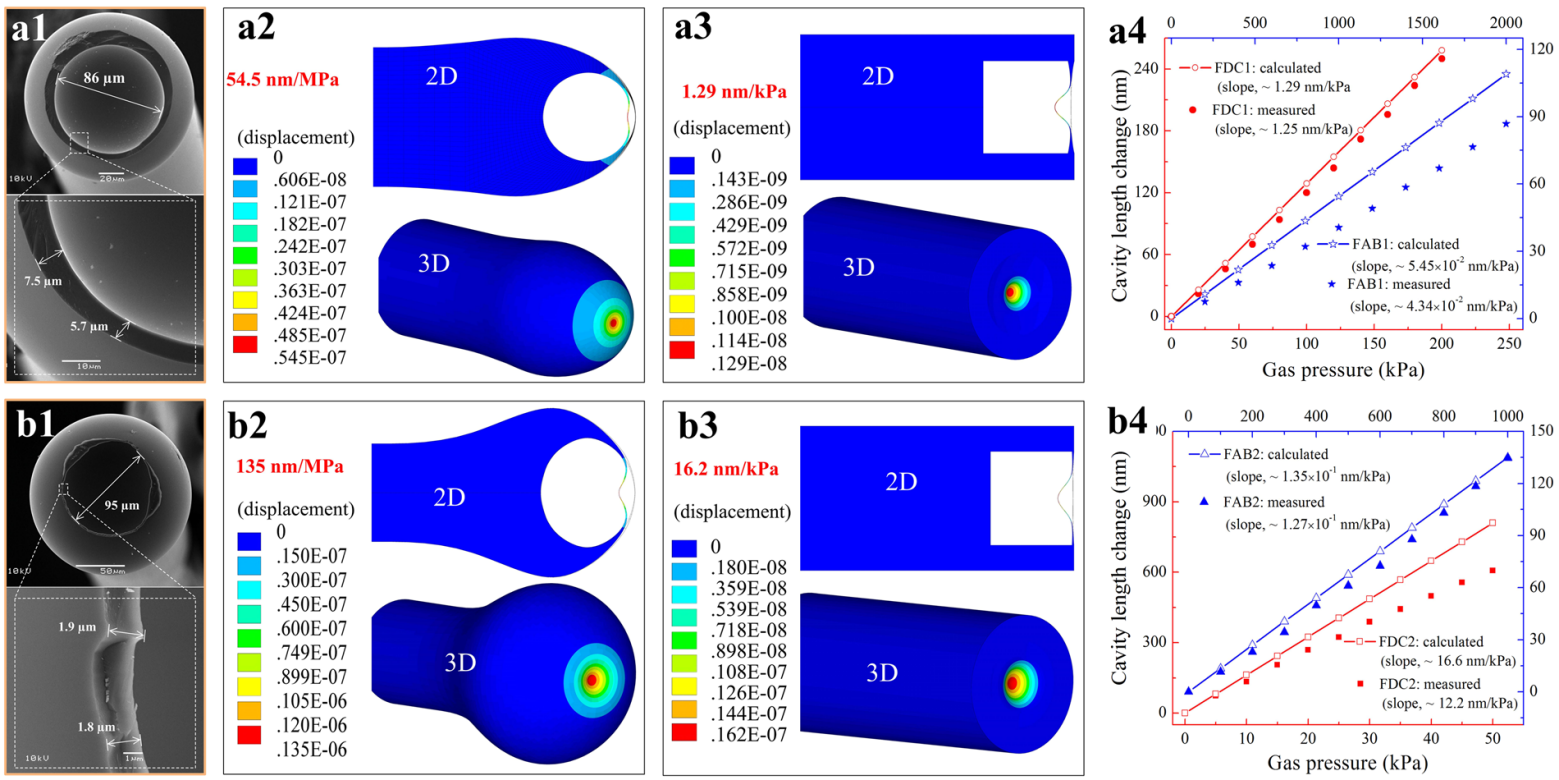
The results of numerical simulation for the cavity length change of two types of specimens employing an FAB or an FDC under an applied pressure. SEM images in (**a1**,**b1**) present the edges of FAB specimens FAB1 and FAB2 exposed by cutting, where the measured dimensions are used for approximating the geometric parameters of the FDC models employed for specimens FDC1 and FDC2, respectively. Simulated deformation contours under an applied gas pressure are presented in (**a2**,**a3,b2**,**b3**), where the red areas represent regions of the sensing diaphragm with the largest deformation. (**a4,b4**) The calculated and measured cavity length changes of the four specimens, FAB1, FDC1, FAB2, and FDC2, versus the applied gas pressure, where the calculated sensitivity are 54.5 × 10^−2^ (), 1.29 (), 1.35 × 10^−1^ () and 12.22 nm/kPa (), respectively; the measured sensitivity are 4.34 × 10^−2^ (), 1.25 (), 1.27 × 10^−1^ () and 16.6 nm/kPa (), respectively.

To investigate the deformation of the sensing diaphragm under an applied gas pressure, the sensor samples above, FAB1, FDC1, FAB2, and FDC2, were modeled using commercial finite element analysis software. The simulations employed standard parameters for silica, i.e., a silica density of 2700 kg/m^3^, Young’s modulus of 73 GPa, and Poisson’s ratio of 0.17. For the purpose of modeling, the geometric dimensions of FAB1 and FAB2 can be obtained from the corresponding optical microscopy images shown in Figs 3(a1) and 5(a1), respectively. The geometric dimensions of FDC1 and FDC2, however, were difficult to obtain directly, and the diaphragm dimensions were approximated. To this end, the diaphragm thicknesses at their edges where they are thickest were first estimated by measuring the thicknesses of FAB edges exposed by cutting, as shown by the SEM images in Fig. 7(a1,b1). Secondly, the thinnest-area thickness at the center of the sensing diaphragms were approximated from the corresponding envelope spacing of the reflection spectra, as shown in Figs 3(b2) and 5(b2). Thirdly, it was assumed that the changing thicknesses of the sensing diaphragms from edge to center conform to a smooth quadric surface. Figure 7(a2,a3,b2,b3) illustrate the two and three-dimensional deformation contours of FAB1, FDC1, FAB2, and FDC2, respectively, where FAB1 and FAB2 are modeled under an applied gas pressure of 1 MPa, and FDC1 and FDC2 are modeled under an applied gas pressure of 1 kPa. Here, the different colors are indicative of the calculated deformation distribution of each specimen. It can be seen that the largest deformations indicated by the color red are located at the centers of the sensing diaphragms. As shown in Fig. 7(a4,b4), the calculated gas pressure sensitivity of FDC1 and FDC2 are 16.2 and 1.29 nm/kPa, respectively, whereas, in contrast, those of FAB1 and FAB2 are 5.45 × 10^−2^ and 1.35 × 10^−1^ nm/kPa, respectively. As such, the calculated gas pressure sensitivity of FDC2 is about 297 times and 120 times greater than those of FAB1 and FAB2, respectively, which agrees well with the measured values of S_p_.

## Conclusions

We have demonstrated a novel fabrication technology for creating an all-silica, nano-scale sensing diaphragm that can be employed for ultrahigh-sensitivity gas pressure sensors based on an FPI employing an FDC. The fabrication technology also achieves the direct thermal splicing of an optical fiber, silica capillary, and nano-scale all-silica diaphragm using only a common fusion splicer. Additionally, to our knowledge, we have achieved the thinnest all-silica diaphragm thus far obtained by means of an electrical arc-discharge technique, with a diaphragm thickness of about 170 nm. Furthermore, the FPI-based gas pressure sensors employing an FDC demonstrate good high-temperature stability and ultrahigh gas pressure sensitivity, where a specimen sensor with a cavity length of about 55 µm exhibited a gas pressure sensitivity of 12.22 nm/kPa, which is more than two orders of magnitude greater than that of FPI-based gas pressure sensors employing an FAB. Moreover, the sensor was measured at high-temperatures of up to 1000 °C, and the ratio of the cavity length variation to the temperature change was about 1300 pm/°C and the temperature-induced gas pressure measurement error was less than 106 Pa/°C. The simple fabrication process, compact size, improved high-temperature stability, and ultrahigh-sensitivity of the proposed FPI-based gas pressure sensor employing an FDC would be eminently suitable for utilization in miniature and highly sensitive pressure, acoustic, and mass sensors for biomedical, environmental, microsystem, and nano-system applications.
